# Nano/Micro Biosensors for Biomedical Applications

**DOI:** 10.3390/bios14020058

**Published:** 2024-01-23

**Authors:** Jin-Ho Lee, Ki Su Kim

**Affiliations:** 1 School of Biomedical Convergence Engineering, Pusan National University, Yangsan 50612, Republic of Korea; 2 Department of Information Convergence Engineering, Pusan National University, Yangsan 50612, Republic of Korea; 3 Department of Convergence Medical Sciences, School of Medicine, Pusan National University, Yangsan 50612, Republic of Korea; 4 School of Chemical Engineering, Pusan National University, Busan 46241, Republic of Korea; 5 Department of Organic Material Science & Engineering, Pusan National University, Busan 46241, Republic of Korea; 6 Institute of Advanced Organic Materials, Pusan National University, Busan 46241, Republic of Korea

## Introduction

Advances in nano/micro technologies in recent years have significantly improved biosensors in terms of their viability for biomedical purposes, from diagnostic to therapeutic applications, allowing for effective early detection and personalized treatment modalities. Specifically, the introduction of a variety of nano/micro technologies has offered new opportunities to improve the sensitivity, selectivity, response time, and biocompatibility of biosensors through outstanding physical, chemical, electrical, and electrochemical properties. This Special Issue aims to highlight the most recent and promising nano/micro technologies utilized in the development of biosensors for biomedical applications. We thus collated 10 original research papers and review articles aligned with these themes, to lead the charge towards new approaches to and solutions for a next-generation biosensor for biomedical applications.

Anara Molkenova et al. (Contribution 1) presents the research trend of plasmon modulated upconversion biosensors. Luminescent behavior UCNPs have been widely utilized for background-free biorecognition and biosensing. Currently, a paramount challenge exists in how to maximize NIR light harvesting and upconversion efficiencies for achieving a faster response and better sensitivity without damaging the biological tissue upon laser-assisted photoactivation. In this article, they offer the reader an overview of the recent updates, exciting achievements, and challenges in the development of plasmon-modulated upconversion nanoformulations for biosensing applications.

Hanbin Park et al. (Contribution 2) reports on the recent advancements in nanomaterial-based microcystin (MC) biosensors. The study addresses the environmental issue of eutrophication in lakes and rivers, where harmful toxins are produced by cyanobacterial algae. The consumption of these contaminants from the water, particularly microcystins, poses serious health risks, increasing the risk, for example, of liver failure and hepatocirrhosis. Recognizing the importance of precise MC detection in water samples, this review highlights the promising developments in nanomaterial-based MC biosensors, emphasizing their potential to overcome the limitations of traditional detection methods.

Hye Kyu Choi et al. (Contribution 3) presents an enzymatic electrochemical/fluorescent nanobiosensor for the detection of small chemicals. As techniques used to implement the sensing function of such enzymatic biosensors, electrochemical and fluorescence techniques have mostly been used for the detection of small molecules because of their advantages. In addition, through the incorporation of nanotechnologies, the detection property of each technique-based enzymatic nanobiosensor can be improved to measure harmful or important small molecules accurately. This review provides interdisciplinary information related to developing enzymatic nanobiosensors for small molecule detection, such as widely used enzymes, target small molecules, and electrochemical/fluorescence techniques.

Jin-Ha Choi (Contribution 4) presents the utilization of functional nanomaterials in the development of proteolytic biosensors. A proteolytic enzyme can be selectively quantified by changing the detectable signals causing the degradation of the peptide chain. In addition, by combining polypeptides with various functional nanomaterials, proteolytic enzymes can be measured more sensitively and rapidly. In this paper, proteolytic enzymes that can be measured using a polypeptide degradation method are reviewed, and recently studied functional nanomaterials-based proteolytic biosensors are discussed

Rowoon Park et al. (Contribution 5) emphasizes the potential use of molecularly imprinted polymer (MIP) in the development of molecularly imprinted polymer (MIP)-based in vitro diagnostic medical devices for point-of-care testing (POCT). The article highlights the potential impact of an artificial bioreceptor, molecularly imprinted polymer (MIP), in the development of POCT devices. Taking advantage of their physicochemical stability, MIP could improve their analytical performance in physiological conditions along with their stability. As such, the article discusses new point-of-care testing (POCT) devices designed to detect biomarkers in clinical biofluids like sweat, tears, saliva, or urine.

Yoo-Kyum Shin et al. (Contribution 6) focus on the micro/nano-structured biodegradable pressure sensors designed for biomedical applications. The growing interest in these sensors arises from their temporal nature in wearable and implantable devices while eliminating biocompatibility concerns. Recent advancements in micro/nano-technologies, including device structures and materials, have significantly enhanced the performance and functionality of biodegradable pressure sensors. The review emphasizes the improved device-level capabilities achieved through adjustments in geometrical design parameters at the micro and nanometer scale. The discussion covers material choices and sensing mechanisms, historical developments in biodegradable pressure sensors, fabrication methods, device performance, and biocompatibility.

Kwang-Ho Lee et al. (Contribution 7) reports the recent advances in multicellular tumor spheroid generation and its applications in drug screening. Multicellular tumor spheroids (MCTs) have been employed in biomedical fields owing to their advantage in designing a three-dimensional (3D) solid tumor model. In drug cytotoxicity assessments, MCTs provide better mimicry of conventional solid tumors that can precisely represent anticancer drug candidates’ effects. To generate and incubate multicellular spheroids, researchers have developed several 3D multicellular spheroid culture technologies to establish a research background and a platform using tumor modeling advanced materials science and biosensing techniques for drug screening. In application, drug screening is performed in both an invasive and non-invasive manner, according to their impact on the spheroids.

Chuntae Kim et al. (Contribution 8) reports the recent trends in exhaled breath diagnosis using an artificial olfactory system. Artificial olfactory systems are needed in various fields that require real-time monitoring, such as healthcare. This review introduces cases of detection of specific volatile organic compounds (VOCs) in a patient’s exhaled breath and discusses trends in disease diagnosis technology development using artificial olfactory technology that analyzes exhaled human breath. By regularly monitoring health status, diseases can be prevented or treated at an early stage, thus increasing the human survival rate and reducing overall treatment costs. This review introduces several promising technical fields to develop technologies that can monitor health conditions and diagnose diseases early by analyzing exhaled human breath in real time.

Svetlana Mescheryakova et al. (Contribution 9) presents an interesting study that developed a fluorescent-based nanosensor for the detection of doxorubicin by utilizing alloyed semiconductor QDs. Owing to their photostability, uniformity, and colloidal stability, CdZnSeS/ZnS core/shell nanocrystals (QD) were successfully employed to develop a nanosensor. The analytical approach exploits changes in emission intensity through the quenching effect, while an interaction occurs between the nanosensors and modulating molecules. To maximize the quenching efficiency, QDs, alloyed CdZnSeS/ZnS core/shell nanocrystals, were carefully modified with thioglycolic acid (TGA) and 3-mercaptopropionic acid (MPA). The developed fluorescence-based turn-off nanosensors were able to determine DOX from undiluted human plasma. The calculated limit of detection values was 0.08 and 0.03 ug/mL, while QDs were stabilized with thioglycolic and 3-mercaptopropionic acids, respectively. As such, recognizing the critical importance and challenges of clinical sample detection, and continuously improving and developing cutting-edge technologies in clinical sample analysis, will help ensure accurate diagnostics for clinical and medical approaches.

Samantha Nunez et al. (Contribution 10) introduces a fluorescent inducible system that can quantify the free fucose in Escherichia coli. As fucose metabolism generates short-chain fatty acids and plays a key role in facilitating cross-feeding microbial interactions, the bacterial consumption of fucose plays a pivotal role in the assembly of the gut microbiome in infants. In this regard, they designed a molecular quantification method for free fucose using fluorescent Escherichia coli. They assessed low- and high-copy plasmids with and without the transcription factor fucR, along with the corresponding fucose-inducible promoter controlling the reporter gene sfGFP. The proposed system was successfully validated through the supernatant of Bifidobacterium bifidum JCM 1254 supplemented with 2-fucosyl lactose.

Overall, though this Special Issue contains several research papers and review articles that highlight different aspects of biosensors and their applications in various fields, we believe each article provides valuable insights into the utilization of nano/micro technologies in the development of biosensors for biomedical applications.

